# Plasma expression of microRNA-425-5p and microRNA-451a as biomarkers of cardiovascular disease in rheumatoid arthritis patients

**DOI:** 10.1038/s41598-021-95234-w

**Published:** 2021-08-02

**Authors:** Delia Taverner, Dídac Llop, Roser Rosales, Raimon Ferré, Luis Masana, Joan-Carles Vallvé, Silvia Paredes

**Affiliations:** 1 Sección de Reumatología, Hospital Universitario Sant Joan, Reus, Catalonia Spain; 2 Unitat de Recerca de Lípids i Arteriosclerosi, Universitat Rovira i Virgili, Reus, Catalonia Spain; 3 Institut D’Investigació Sanitària Pere Virgili (IISPV), Reus, Catalonia Spain; 4 Centro de Investigación Biomédica en Red de Diabetes y Enfermedades Metabólicas Asociadas, Madrid, Spain; 5 Servicio de Medicina Interna, Hospital Universitario Sant Joan, Reus, Catalonia Spain; 6 Facultat de Medicina, Universitat Rovira i Virgili, Sant Llorenç 21, 43201 Reus, Catalonia Spain

**Keywords:** Cardiology, Diseases, Rheumatology, Risk factors

## Abstract

To validate in a cohort of 214 rheumatoid arthritis patients a panel of 10 plasmatic microRNAs, which we previously identified and that can facilitate earlier diagnosis of cardiovascular disease in rheumatoid arthritis patients. We identified 10 plasma miRs that were downregulated in male rheumatoid arthritis patients and in patients with acute myocardial infarction compared to controls suggesting that these microRNAs could be epigenetic biomarkers for cardiovascular disease in rheumatoid arthritis patients. Six of those microRNAs were validated in independent plasma samples from 214 rheumatoid arthritis patients and levels of expression were associated with surrogate markers of cardiovascular disease (carotid intima-media thickness, plaque formation, pulse wave velocity and distensibility) and with prior cardiovascular disease. Multivariate analyses adjusted for traditional confounders and treatments showed that decreased expression of microRNA-425-5p in men and decreased expression of microRNA-451 in women were significantly associated with increased (β = 0.072; *p* = 0.017) and decreased carotid intima-media thickness (β = −0.05; *p* = 0.013), respectively. MicroRNA-425-5p and microRNA-451 also increased the accuracy to discriminate patients with pathological carotid intima-media thickness by 1.8% (*p* = 0.036) in men and 3.5% (*p* = 0.027) in women, respectively. In addition, microRNA-425-5p increased the accuracy to discriminate male patients with prior cardiovascular disease by 3% (*p* = 0.008). Additionally, decreased expression of microRNA-451 was significantly associated with decreased pulse wave velocity (β = −0.72; *p* = 0.035) in overall rheumatoid arthritis population. Distensibility showed no significant association with expression levels of the microRNAs studied. We provide evidence of a possible role of microRNA-425-5p and microRNA-451 as useful epigenetic biomarkers to assess cardiovascular disease risk in patients with rheumatoid arthritis.

## Introduction

Patients with rheumatoid arthritis (RA) present an increased risk of cardiovascular (CV) disease, estimated to be approximately 50% greater when compared to the general population. This risk is in part due to the inflammatory activity of RA, which plays an important role in the development of atherosclerosis^1,2^. CV disease represents the major cause of morbi-mortality in RA patients, and acute myocardial infarction (AMI) is the most prevalent in those patients^2^. In RA patients, subclinical atherosclerosis (determined by ultrasound carotid intima-media thickness (cIMT) and carotid plaque presence (cPP)) as well as arterial stiffness (measured by pulse wave velocity (PWV) and distensibility) have been accepted as surrogate markers of CV disease with good prediction of CV events^3,4^.

MicroRNAs (miRs) are a family of small, non-coding RNA molecules of approximately 21–25 nucleotides that regulate gene expression at the post-transcriptional level^5^. miRs possess excellent stability in plasma, and circulating miRs have great potential as disease biomarkers. They have been implicated in many biological processes, including autoimmune diseases, as they are able to modulate adaptative responses and the differentiation of B and T cells^6^.

Furthermore, abnormal expression of circulating miRs in patients with RA is well documented. Some miRs have been associated with a higher risk and progression to RA^7^ as well as with clinical variables of RA [tender joint and disease activity score-erythrocyte sedimentation rate (DAS28-ESR)]^8,9^. In addition, miRs are recognized as critical regulators in atherosclerosis^10^, some specific miRs are useful for the early detection of AMI, and differential expression of miRs has been identified in patients with coronary artery disease and atherosclerosis^11–14^. However, the association of miRs with cardiovascular disease in patients with RA remains unclear^15^. In a previous discovery study^16^ carried out in male subjects, we found 10 plasma miRs that were expressed at similar levels in patients with RA and with acute myocardial infraction (AMI) and different from healthy controls, which were candidates as biomarkers of CV disease in RA patients for the present study. Of these 10 miRs, six (miR Let-7a, miR-96, miR-381, miR-425-5p, miR-451, and miR-572) were included for validation in the present study and four miRs were discarded due to very low-level expression.

Thus, the objective of the present study was to validate in independent plasma samples (214 RA patients – validation cohort) whether those six miRs are associated with surrogate markers of CV disease and so can facilitate an early diagnosis of CV disease in RA patients.

## Results

### Characteristics of RA cohort

We included 214 patients with RA in the study. General characteristics of the cohort are shown in Table 1 and in previous publications^17,18^. Briefly, the mean age and disease onset were 58 (12) and 9.4 (9.1) years, respectively. Female patients represented 64.5% of the cohort; 60% of patients were hypertensive, 11.7% were diabetic, 41% were dyslipidaemic, and 26% were smokers. The percentages of patients in remission or with low, moderate or high disease activity were 27, 19, 44, and 10, respectively. There were differences between men and women in some of these parameters (Table 1 and previous publication^18^). Disease-modifying antirheumatic drugs were administered to 95% of the patients, which included 75% receiving non-biological drugs and 20% biological drugs. Fifty-seven percent of patients received non-steroidal anti-inflammatory drugs, and/or 51% received corticosteroids.

**Table 1:** Description of general characteristics, Disease features, and treatments of RA patients overall and stratified by gender.

	RA (n = 214)	Female (n = 138)	Male (n = 76)	*P*
**Characteristics of the groups**				
Gender-female (%, n)	64.5 (138)			
Age (years, SD)	58(12)	57 (12)	59 (12)	0.55
Body mass index (kg/m^2^, SD)	27.8 (5.9)	27.7 (6.6)	28.1 (4.4)	0.62
Waist circumference (cm, SD)	93 (15)	88 (15)	100 (12)	< 0.001
SBP (mmHg, SD)	137 (21)	135 (21)	142 (21)	0.024
DBP (mmHg, SD)	81 (12)	80 (13)	84 (12)	0.025
LDL cholesterol (mg/dL, SD)	119 (31)	118 (31)	120 (31)	0.75
HDL cholesterol (mg/dL, SD)	66 (19)	72 (18)	54 (15)	< 0.001
Triglycerides (mg/dL, SD)	105 (55)	102 (54)	112 (58)	0.21
Glucose (mg/dL, SD)	95 (23)	94 (25)	96 (18)	0.35
Current smoker (%, n)	26.2(56)	27.5(38)	23.7(18)	0.34
Hypertension (%, n)	60.3 (129)	53 (73)	74 (56)	0.003
Diabetes mellitus (%, n)	11.7 (25)	10.9 (15)	13.2 (10)	0.62
Dyslipidaemia (%, n)	41.1(88)	39.1 (54)	44.7 (34)	0.425
**Disease features**				
Disease onset (years, SD)	9.4 (9.1)	10.1 (9.9)	8.2 (7.5)	0.12
DAS28 (%, n)	3.5 (1.3)	3.7 (1.3)	2.98 (1.1)	< 0.001
Remission (%, n)	27.1 (58)	20.3 (28)	39.5 (30)	< 0.001
Low activity (%, n)	18.7 (40)	14.5 (20)	26.3 (20)	
Moderate activity (%, n)	44.4 (95)	52.2 (72)	30.3 (23)	
High activity (%, n)	9.8 (21)	13 (18)	3.9 (3)	
HAQ (mean, SD)	0.45 (0.52)	0.59 (0.56)	0.21 (0.34)	< 0.001
Rheumatoid factor + (%, n)	72.4 (155)	71.7 (99)	73.7 (56)	0.76
ACPA + (%, n)	81.3 (174)	83.3 (115)	77.6 (59)	0.31
ESR (mm/h, SD)	37 (26)	40 (27)	32 (22)	0.019
CRP (mg/dL, SD)	0.72 (0.83)	0.73 (0.80)	0.70 (0.88)	0.83
Fibrinogen (mg/dL, SD)	443 (97)	442 (96)	444 (100)	0.91
**Treatments (%, n)**				
DMARDs	75.2 (161)	71.7 (99)	81.6 (62)	0.11
Biological agent	20.1 (43)	23.2 (32)	14.5 (11)	0.13
NSAIDs	57 (122)	57.2 (79)	56.6 (43)	0.92
Corticosteroids	50.9 (109)	53 (73)	47 (36)	0.44

n = number of individuals, SBP = systolic blood pressure, DBP = diastolic blood pressure, HAQ = health assessment questionnaire index, ACPA = citrullinated anti-cyclic peptide antibodies, ESR = erythrocyte sedimentation rate, CRP = C-reactive protein, DAS28 = disease activity score, DMARDs = disease-modifying antirheumatic drugs, NSAIDs = non-steroidal anti-inflammatory drugs, SD = standard deviation, *p* = *p* value.

### Associations of candidate miRs with cIMT, cPP and prior cardiovascular disease

**Figure 1:**
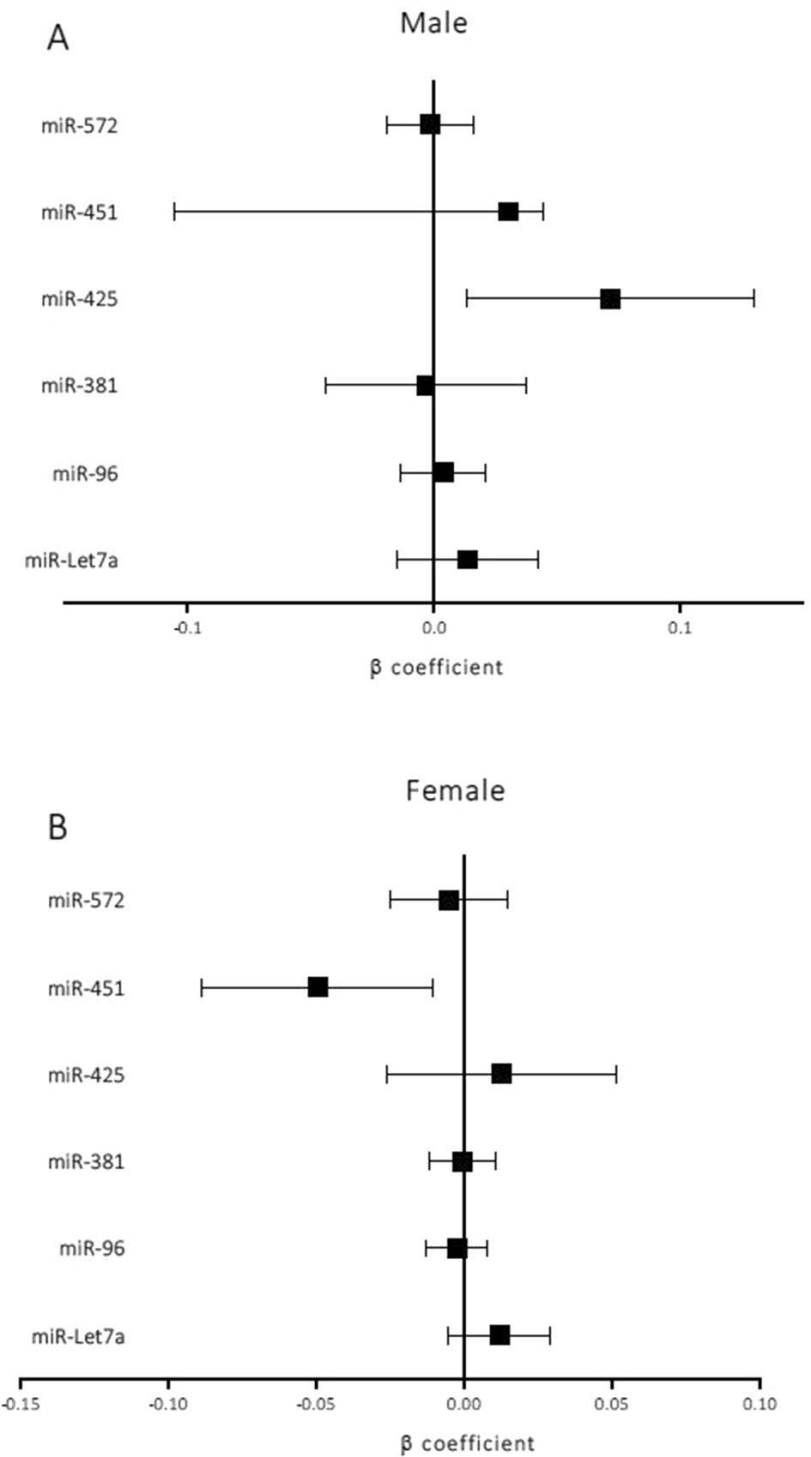
Adjusted β linear regression estimates with 95% confidence intervals of the effect on cIMT of a ΔCt increase (decreased expression) of the different miRs applied to men and women separately. The models are adjusted for RA disease onset, body mass index, age, ischaemic heart disease, ictus, peripheral artery disease, creatinine, hypertension, dyslipidaemia, type 2 diabetes mellitus, disease-modifying antirheumatic drugs, biological agents, corticosteroids, and non-steroidal anti-inflammatory drugs.

We first evaluated the correlations of the candidate miRs^16^ with cIMT stratified by sex according to the previous interaction that we observed between age and sex^18^ (see Methods). Univariate analyses showed that only miR-451 was significantly associated with cIMT in women (Supplementary Table 1). No miRs were associated with cIMT in men. However, when adjusted for age (Supplementary Table 2), we observed that the levels of miR-425-5p in men and miR-451 in women were associated with cIMT. Furthermore, after adjusting for traditional confounders and treatments (Fig. 1), multivariable linear regression analyses showed that the expression levels of both miRs were independent predictors of cIMT. Specifically, decreased expression of miR-425-5p was significantly associated with increased cIMT in men (β = 0.072; *p* = 0.017) (Fig. 1A), and miR-425-5p expression level contributed a significant 6% (R^2^ change in Supplementary Fig. 1) to the explanation of cIMT variability, with the overall model explaining 61% (R^2^ in Supplementary Fig. 1) of the cIMT variability in men. Decreased expression of miR-451 was significantly associated with decreased cIMT in women (β = −0.05; *p* = 0.013) (Fig. 1B), and miR-451 expression level provided a significant extra 3.5% (R^2^ change in Supplementary Fig. 1) of the explanation of cIMT variability, with the overall model explaining 38% (R^2^ in Supplementary Fig. 1) of the cIMT variability in women. The cIMT predicted for men and women had a highly significant correlation with the cIMT observed (r = 0.78; *p* = 0.005 for men and r = 0.62; *p* = 0.011 for women) (Supplementary Fig. 2). Then, to check the heterogeneity of our population, we added interaction terms between miR-425-5p and miR-451 and disease activity, inflammatory characteristics and treatment variables to the models. We observed no significant effects of these terms on the variability of cIMT explained by the models (Supplementary Table 3). The expression of the other miRs selected (miR-Let7a, miR-96, miR-381, and miR-572) was not associated with cIMT in men or women (Fig. 1A,B). As for cPP none of the miRs studied contributed significantly to the explanation of the variability of cPP (Supplementary Table 4).

**Figure 2:**
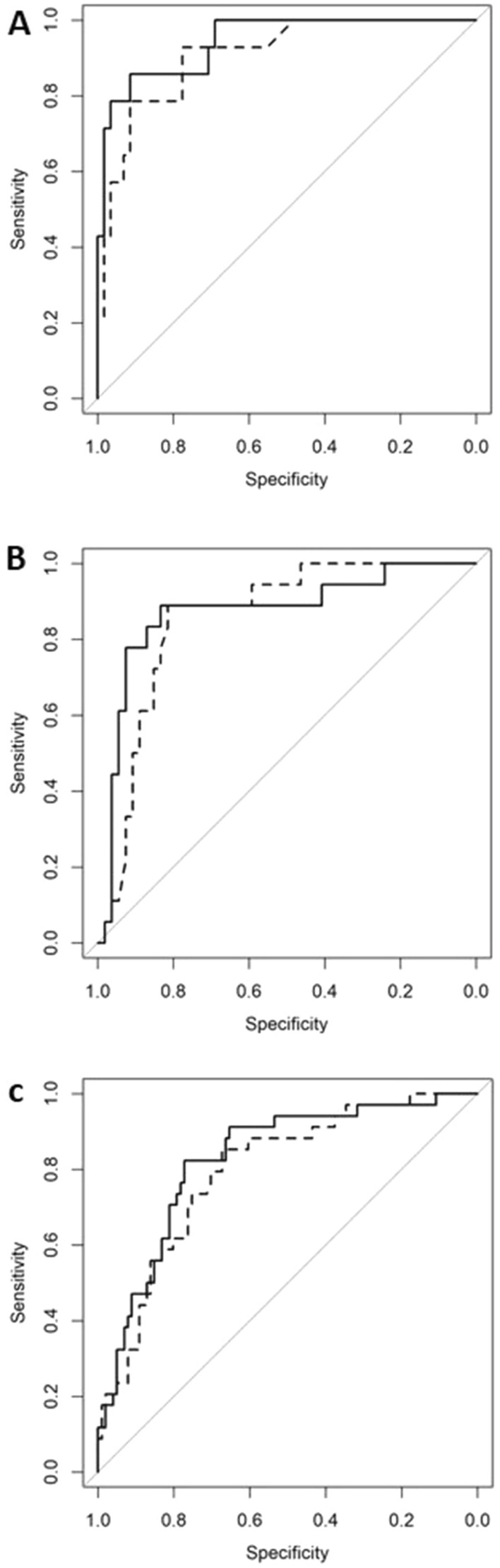
ROC curves for the multivariable logistic regression models with or without candidate miRs expression. (**A**) Estimation of prior CVD in men. (**B**) Estimation of pathological cIMT in men. (**C**) Estimation of pathological cIMT in women. Models were assessed by logistic regression analysis and were initially adjusted for age, RA disease onset, BMI, ischemic heart disease, peripheral artery disease, ictus, creatinine, and treatments. Solid line = baseline models. Dotted line = baseline model + candidate miRs.

However, multivariable logistic regression models showed that the miR-425-5p and miR-451 expression levels were able to significantly predict pathological cIMT in men (*p* = 0.036) and women (*p* = 0.021), respectively (Table 2). In addition, adding miR-425-5p or miR-451 expression to each basal model significantly increased the predictive ability of the model as the area under the receiver operating characteristics (ROC) curve (AUC) increased by 1.83% in men and 3.56% in women for miR-425-5p (*p* = 0.03) and miR-451 (*p* = 0.013), respectively (Fig. 2 and Table 3). According to the Youden index, the optimal cut-off points for the final models were 0.26 and 0.246 for miR-425-5p and miR-451, respectively, and for these cut-off points, Table 3 shows the AUC, specificity, sensitivity, positive predictive value (PPV), negative predictive value (NPV), and likelihood ratio test of the different models adjusted. Interestingly, the miR-425-5p expression levels were able to significantly increase the accuracy to discriminate male RA patients who have had prior CVD. Thus, after adjusting the regression logistic models, the miR-425-5p expression levels were significantly associated with prior CVD (*p* = 0.008) (Table 2). Moreover, the AUC of the ROC curves showed that the addition of miR-425-5p to the basal model increased the accuracy of the discrimination by 3% (*p* = 0.008) (Fig. 2 and Table 3) with an optimal cut-off point of 0.35 and a sensitivity, specificity, PPV, and NPV of 0.79, 0.97, 0.85, and 0.95, respectively (Table 3). In addition, the mean decrease Gini plot from the random forest analysis showed that miR-425-5p was the most important variable in terms of the discrimination ability to classify male patients with prior CVD from those without (Supplementary Fig. 3).

**Table 2:** Adjusted OR estimates of the effect of miR-425-5p and miR-451 expression on pathological cIMT (A) and on prior CVD (B).

	OR	*P*	R^2^ (%)	AIC
**A. Pat-cIMT**				
Model 1			22	129.52
Model 1 + miR-451	0.088	0.017	28	126.33
Model 2			29	70.23
Model 2 + miR-425-5p	4.95	0.06	39	67.87
**B. Prior CVD**				
Model 3			42	54.43
Model 3 + miR-425-5p	17.06	0.026	53	49.4

Adjusted OR estimates were assessed by logistic regression analysis applied to women (model 1) and to men (model 2 and 3) with or without inclusion of candidate miRs expression. Models were initially adjusted for age, RA disease onset, BMI, ischemic heart disease, peripheral artery disease, ictus, creatinine, and treatments. OR = odds ratio, AIC = Akaike information criteria. *p* = *p* value.

**Table 3:** ROC analysis parameters of pathological cIMT (A) and prior CVD (B) logistic models.

	AUC	Cut-off	Sensitivity	Specificity	PPV	NPV	*P*
**A. Pat-cIMT**							
Model 1	0.8	0.21	0.85	0.67	0.47	0.93	
Model 1 + miR-451	0.83	0.19	0.91	0.7	0.51	0.96	0.027
Model 2	0.85	0.26	0.89	0.81	0.62	0.96	
Model 2 + miR-425-5p	0.87	0.24	0.83	0.89	0.64	0.96	0.036
**B. Prior CVD**							
Model 3	0.91	0.16	0.93	0.78	0.5	0.98	
Model 3 + miR-425-5p	0.93	0.35	0.97	0.79	0.85	0.95	0.008

ROC analyses were performed by logistic regression analysis applied to women (model 1) and to men (model 2 and 3) with or without inclusion of candidate miRs expression. Models were initially adjusted for age, RA disease onset, BMI, ischemic heart disease, peripheral artery disease, ictus, creatinine, and treatments. *P* values were obtain from the Likelihood Ratio test. AUC = area under the curve. PPV = positive predicted value. NPV = negative predicted value. *P* = *p* value.

### Associations of candidate miRs with PWV and distensibility

**Figure 3:**
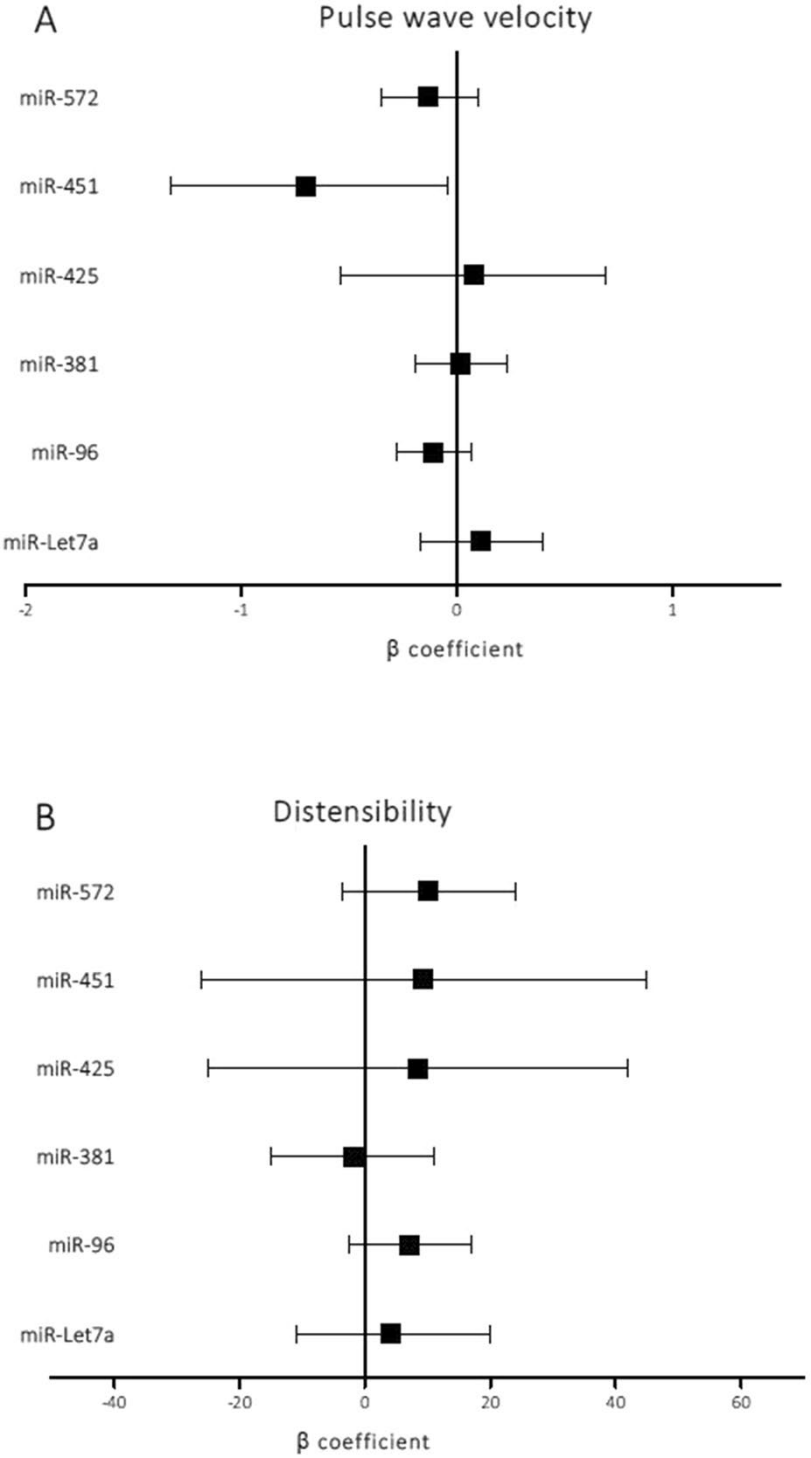
Adjusted β linear regression estimates with 95% confidence intervals of the effect on PWV and distensibility of a ΔCt increase (decreased expression) of the different miRs applied to overall population. The models were adjusted for RA disease onset, body mass index, age, ischaemic heart disease, ictus, hypertension, dyslipidaemia, type 2 diabetes mellitus, disease-modifying antirheumatic drugs, biological agents, corticosteroids, and non-steroidal anti-inflammatory drugs.

Regarding stiffness markers, univariate analysis in the overall population did not show an association of any candidate miR with PWV. However, when adjusting for traditional confounders and treatments, we observed that decreased expression of miR-451 was significantly associated with decreased PWV (β = − 0.72; *p* = 0.035) (Fig. 3A). Although the effect size was small (R^2^ change = 1.6%), miR-451 expression level significantly contributed to the explanation of PWV variability, with the overall model explaining 36% of that variability. Furthermore, the interaction terms between miR-451 and disease activity, inflammatory characteristics and treatments variables did not significantly affect the variability of PWV explained by the models (Supplementary Table 3). The expression of miR-425-5p, miR-Let7a, miR-96, miR-381, or miR-572 was not associated with PWV. None of the candidate miRs showed a significant association with distensibility (Fig. 3B).

### Variables associated with the expression levels of miRs

Next, we evaluated which variables were significantly associated with the expression levels of miR-425-5p and miR-451. We tested, in the overall population, disease-related variables and variables characteristics of the population studied. We observed that the expression of miR-425-5p was negatively correlated with ESR (r = − 0.136; *p* = 0.048) and that the expression of miR-451 was positively correlated with DAS28 (r = 0.19; *p* = 0.006), ESR (r = 0.23; *p* = 0.001), CRP (r = 0.15; *p* = 0.033), and fibrinogen (r = 0.28; *p* = 0.0001). Those correlations were basically maintained in men, while in women, only fibrinogen was correlated with the expression of miR-451 (r = 0.175; *p* = 0.041) (Table 4).

**Table 4:** Disease-related variables and variables characteristics of the population studied associated with the expression levels of miR-425-5p and miR-451 stratified by sex.

	DCt-425			DCt-425		
	r	β	*p*	r	β	*p*
**Men**						
Disease duration	0.092	0.006	0.434	0.06	0.004	0.609
HAQ	− 0.022	− 0.031	0.853	− 0.001	− 0.001	0.993
DAS28	− 0.134	− 0.06	0.255	0.284	0.120	0.014
ESR	− 0.244	− 0.005	0.036	0.385	0.008	0.001
CRP	− 0.156	− 0.085	0.185	0.148	0.077	0.208
Fibrinogen	− 0.150	− 0.001	0.203	0.435	0.002	0.0001
Age	− 0.56	− 0.002	0.635	0.0076	0.003	0.52
BMI	0.002	0.0001	0.984	0.194	0.02	0.097
SBP	0.076	0.002	0.521	0.146	0.003	0.216
DBP	0.028	0.001	0.810	0.069	0.003	0.558
Glucose	0.024	0.001	0.840	0.016	0.0001	0.891
HbA1c (%)	− 0.081	− 0.064	0.497	− 0.01	− 0.008	0.932
**Women**						
Disease duration	0.091	0.004	0.29	0.036	0.001	0.680
HAQ	0.109	0.086	0.203	0.127	0.096	0.138
DAS28	0.107	0.035	0.215	0.163	0.051	0.056
ESR	− 0.091	− 0.001	0.290	0.161	0.002	0.06
CRP	− 0.038	− 0.02	0.662	0.147	0.075	0.087
Fibrinogen	− 0.041	0.0001	0.630	0.175	0.001	0.041
Age	− 0.045	− 0.002	0.602	0.049	0.002	0.566
BMI	0.008	0.001	0.922	0.04	0.002	0.643
SBP	− 0.118	− 0.002	0.168	− 0.043	− 0.001	0.613
DBP	− 0.056	− 0.002	0.514	0.668	0.002	0.431
Glucose	0.082	0.001	0.328	0.084	0.001	0.331
HbA1c (%)	0.059	0.027	0.493	0.089	0.038	0.303

SBP = systolic blood pressure, DBP = diastolic blood pressure, HAQ = health assessment questionnaire index, ESR = erythrocyte sedimentation rate, CRP = C-reactive protein, DAS28 = disease activity score, BMI = Body mass index, HbA1c = Glycated haemoglobin. r = Pearson’s coefficient, *p* = *p* value, β = linear regression estimates.

### Biological functional analyses

**Figure 4:**
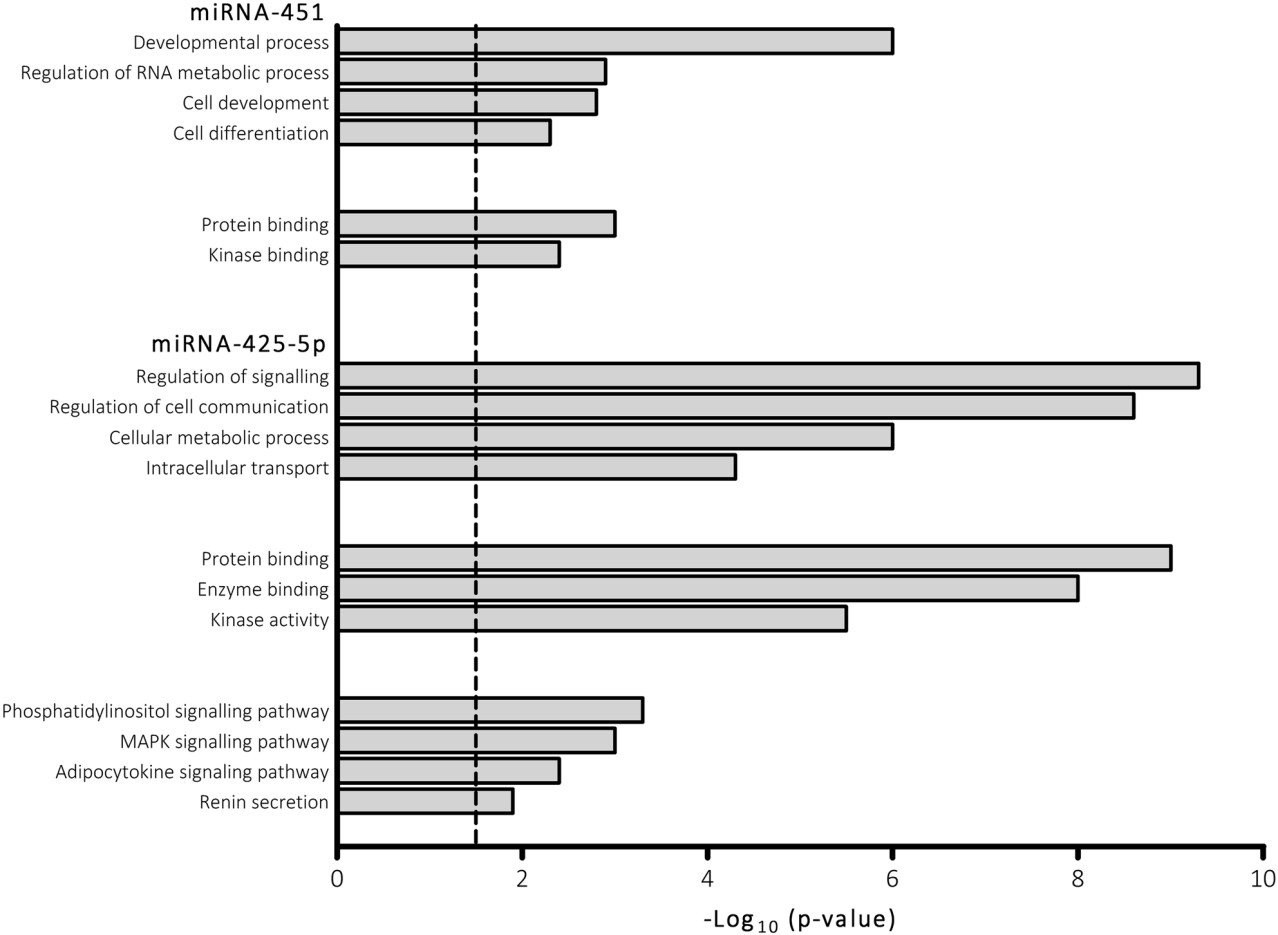
Enrichment analysis of target predicted genes of miR-425-5P and miR-451. Significant biological processes and molecular functions are showed. Functional miR targets were selected using mirDIP v1.4 database and functional classification was analysed with terms from Gene Ontology and pathways from the Kyoto Encyclopaedia of Genes and Genomes.

To address the functional implications of miR-425-5p and miR-451, we predicted their possible target genes using the mirDIP v1.4 database. We identified 2140 and 451 genes as functional targets of miR-425-5p and miR-451, respectively. The GO functional enrichment analysis (Fig. 4) showed that the list of target genes of miRNA-425 was significantly enriched in genes engaged in biological processes, such as regulation of signalling (*p* = 4.9 E−10), regulation of cell communication (*p* = 2.4 E−9), cellular metabolic process (*p* = 6.7 E−7), and intracellular transport (*p* = 2.9 E−5). Regarding the molecular functions, the list of genes was significantly overrepresented in protein binding (*p* = 1 E−9), enzyme binding (*p* = 1 E−8), kinase activity (*p* = 3.6 E−6), etc. The KEGG pathway analysis showed that the genes were involved in phosphatidylinositol signalling (*p* = 3.2 E−4), MAPK signalling pathway (*p* = 8.9 E−4), adipocytokin signalling pathway (*p* = 3.3 E−3) and renin secretion (*p* = 1.13 E−2). Regarding miRNA-451, the target genes were significantly enriched in biological processes, such as regulation of cellular metabolic process (*p* = 2.2 E−6), developmental process (*p* = 6.2 E−7), cell differentiation (*p* = 5.9 E−5) and regulation of RNA metabolic process (*p* = 9.1 E−4), and molecular functions, such as protein and kinase binding (*p* = 7.7 E−4 and *p* = 4.6 E−3, respectively). The KEGG pathway analysis showed no significant results.

## Discussion

**Figure 5:**
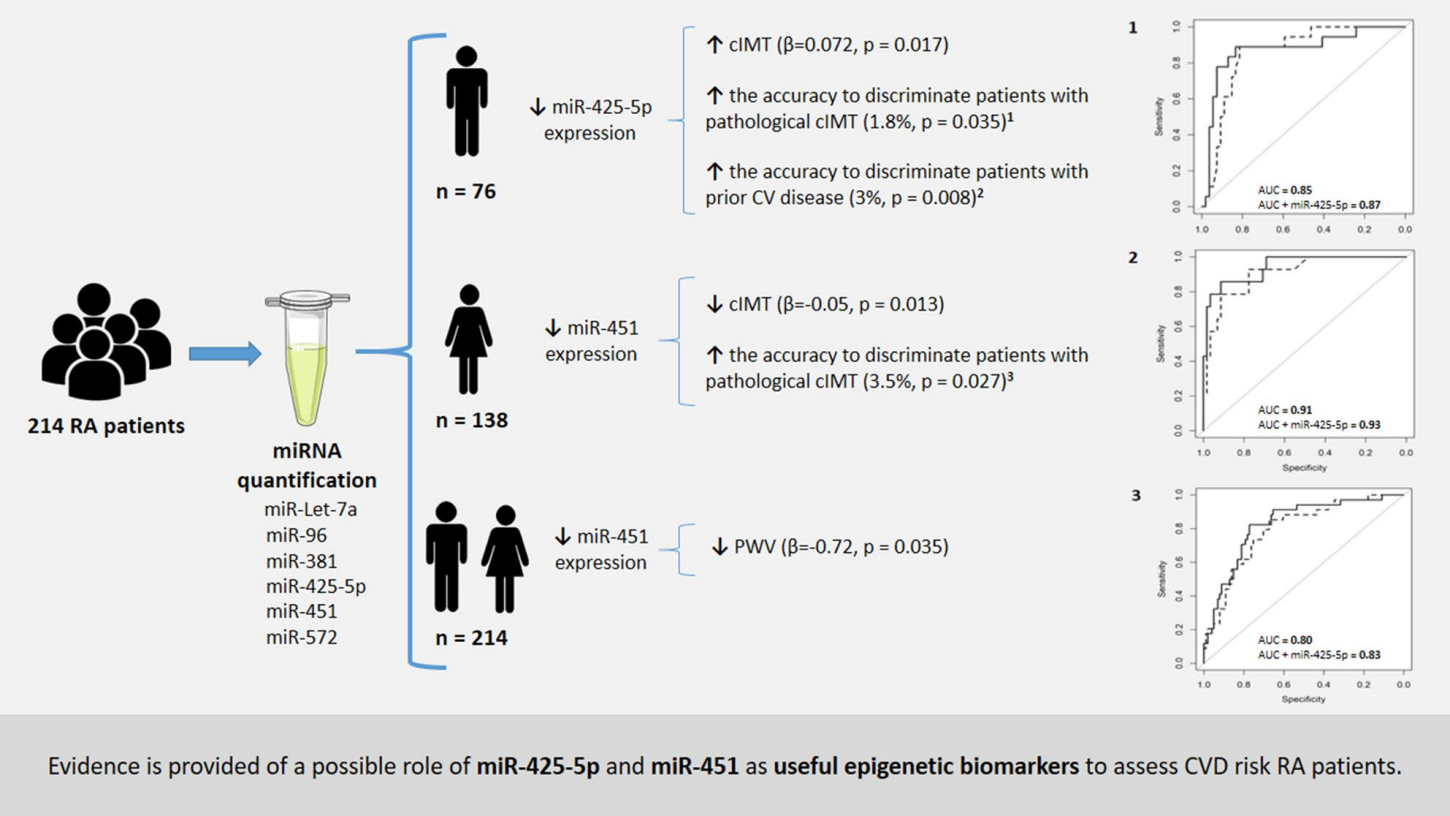
Overview of the main results of the study.

In the present study, we have shown that miR-425-5p and miR-451 are associated, differentially between men and women with RA, with surrogate markers of CV disease such as cIMT and arterial stiffness. Specifically, we observed that in men, a decrease in the plasma expression levels of miR-425-5p was associated with a significant increase in cIMT. The present study is the validation of a previous discovery study in which we evaluated the plasma expression profile of 754 miRs in 7 male patients with RA, in whom 10 miRs were expressed at levels similar to those of 7 male patients with AMI and were downregulated compared their levels with 7 male controls^16^. These results suggested that reduced expression levels of these candidate miRs could indicate a higher CV risk in patients with RA. In the present study carried out in a validation cohort of 214 patients, we validated miR-425-5p, showing that reduced values of expression of this miR were associated with a significant increase in cIMT that could be associated with a higher CV risk in patients with RA. In this sense, we showed that miR-425-5p increased the accuracy to discriminate male patients with prior CVD. In addition, the specificity of action of the miRs has allowed us to identify miR-451 as a new biomarker of CV risk in RA patients. We determined that decreases in the expression of miR-451 showed a protective effect, as they were significantly associated, in women, with lower values of cIMT and, in the entire RA population, with significant decreases in arterial stiffness measured by the PWV. Overall, our results suggest that measuring the expression levels of miR-425-5p and miR-451 in plasma would have clinical interest, since it would identify those RA patients most likely to develop CVD (Fig. 5). However, prospective studies are needed to further validate our proposal. Furthermore, interaction analyses shown that the associations observed between the expression of candidate miRs and cIMT and PWV did not change as a function of disease activity, inflammatory characteristics and treatment variables showing that there is no interference from sample heterogeneity in the associations found in our study.

Many studies have shown that both cIMT and PWV are important and sensitive surrogate markers of CV disease. Different publications have shown an increase in cIMT in RA patients compared with controls^19–22^. In addition, cIMT is able to prospectively predict clinical CV disease events independently of traditional risk factors in CV disease and RA patients^23,24^. In particular, cIMT > 0.9 has a high predictive power for the development of CV events over a 5-year follow-up period^20,25^. Furthermore, PWV, a biomarker of arterial stiffness that reflects early effects on the arterial wall, has been reported to be consistently increased in RA patients^26,27^ and has been shown to independently predict CV events and mortality^26,28^.

There is important evidence in the literature showing that different miRs play pivotal roles in the pathophysiology of RA and CV disease^16^. However, the data describing miRs significantly associated with CV risk in RA patients is limited. Few studies have pointed out the importance of dysregulation of miR expression as directly involved in the pathogenesis of CV disease in patients with RA but without significant results^15,29^. miR-425-5p is mainly associated with neoplasic pathology, although recent data showed that expression levels of this miR improved the prediction of coronary artery calcium in patients with RA^30^. However, the results of our study are the first to associate low expression of this miR with the development of subclinical arteriosclerosis in patients with RA. Furthermore, significantly low levels of miR-425-5p have also been recently described in RA patients compared with controls^31^. Moreover, miR-425-5p has been shown to function as a negative regulator of cardiac fibrosis, and its plasma level has been proposed as a biomarker to predict cardiac fibrosis and heart failure^32^. By contrast, miR-451 has been widely studied in association with RA pathogenesis in RA patients and with atherosclerosis in CVD patients. Our study is the first to associate low expression of this miR with less CV atherosclerosis and arterial stiffness in RA patients. Dysregulation of miR-451 expression has been described in CVD patients. Indeed, miR-451 is highly expressed during myocardial infarction^33^ and played a critical role in cardiac hypertrophy^34^. In addition, data have shown overexpression of miR-451 in patients with coronary artery disease^35,36^, which may modulate the production of pro-inflammatory cytokines. Treatment with statins could decrease the level of this miRNA, making it a potential new biomarker that assesses the efficacy of statins in patients with unstable angina^37^.

Deregulation of miR-451 expression has also been involved in RA pathogenesis. Thus, miR-451 is upregulated in T cells of peripheral blood from RA patients^38^, although downregulation in RA neutrophils has also been described^39^. In addition, miR-451 inhibits the proliferation of synovial fibroblasts and the production of cytokines from patients with RA, so it may be considered a future therapy in RA^40^. Furthermore, in experimental studies in rats, it has been postulated as a possible new biomarker of disease activity and response to treatment^41^.

The differences observed in our study between men and women could have different explanations. The prevalence of traditional cardiovascular risk factors is different between men and women^42^. Furthermore, disease activity may affect the burden of atherosclerosis^43^ and it has been described that measures of disease activity seem to be worse in women than in men^44^. In addition, sex hormones may also be the basis for the differences, as it has been shown that estrogens decrease the inflammatory immune response^45^ and hormone replacement therapy shows a beneficial effect on RA disease activity^46^.

We also observed that plasma expression of miR-425-5p correlated negatively with ESR, while miR-451 correlated positively with DAS28, ESR, CRP and fibrinogen. These unexpected correlations probably indicate that the effects of miR-425-5p and miR-451 on cIMT and PWV observed in this study are not mediated through the classical inflammatory and RA disease activity parameters but through other mediators and are modulated by genetic factors. In addition, we have shown that in our RA population, cIMT was not associated with inflammatory or serological variables^18^. Likewise, the association between miRs and inflammation or RA disease activity has been described in the literature, although not with conclusive results. Thus, of the multiple miRs associated with RA, few have shown a significant relationship with inflammation and disease activity. For instance, miR-146a, which has been described to be overexpressed in the peripheral blood of patients with RA, positively correlates with ESR, while other miRNAs, such as miR-16^9^ and miR-233, have been correlated to DAS28 and the latter also with CRP in patients with initial RA^47^.

Circulating plasma miRs can originate from an active cellular action but they can also reflect altered cellular processes that release these miRs to the circulation. The origin of miR-451 and miR-425-5p has not been addressed in our study. However, we show that passively expression from erythrocytes is not the main source of the plasma levels of miR-425-5p and miR-451 because no significant haemolysis was found in the samples. We believe that plasma levels of miR-425 and miR-451 are provided by other tissues that either actively or passively release these miRs to the circulation.

The differential effect of miR-425-5p and miR-451 on cIMT suggests a high specificity of action of these miRs, and one can speculate that low concentrations of these miRs would be associated with an inability to inhibit the expression of their target gene(s), which would therefore be over-expressed and affect arteriosclerosis development, positively for miR-451 and negatively for miR-425-5p. There are few data in the literature addressing the functional effect of these miRs. We predicted many gene targets for miR-425-5p, the majority of which are involved in signalling pathways such as MAPK, Adipocitokine and PI3K, cell communication processes and binding functions. On the other hand, the predicted gene targets for miR-451 are involved in kinase binding function and in metabolic and cell differentiation processes.

In the present study, the plasma levels of miR-425-5p and miR-451 did not explain the presence of arteriosclerotic plaque in our RA patients. We must consider that cIMT and the presence of arteriosclerotic plaques reflect different stages and features of the arteriosclerosis process^48,49^. Thus, cIMT represents the thickening of the muscular layer of the arterial media layer, while plaque formation is the result of thickening of the arterial intima. In addition, the formation of arteriosclerotic plaques is considered a later stage in the arteriosclerotic process^50^, so the expression levels of the miRs identified in this study could be markers of earlier stages of the arteriosclerotic process, which is relevant to the objective of the present study. In this sense, we showed that miR-425-5p expression was able to predict male patients with prior CVD. Furthermore, variables expressed as function of time, such as cIMT or PWV progression would more accurately predict CV disease development than cross-sectional measurements and might explain the lack of concordance in the association of miR-425-5p with cIMT and PWV.

In conclusion, the results of our study demonstrate that decreases in the expression of miR-425-5p in men and miR-451 in women are associated with higher and lower values ​​of subclinical arteriosclerosis, respectively. Furthermore, decreases in the expression of miR-451 are associated with lower arterial stiffness in the overall RA population. This study provides evidence of a possible role of miR-425-5p and miR-451 as useful epigenetic biomarkers to assess CV risk in patients with RA.

## Methods and patients

### Patients

The present study is the validation of a previous discovery study designed to find new miRs as biomarkers of early CV disease in RA patients^16^. The RA population of the present study has been described in a previous paper^18^. Briefly, the 1987 American College of Rheumatology criteria for RA diagnosis were used to select patients who attended the University Hospital Sant Joan de Reus via external consultation. 214 patients between 20 and 80 years of age were included in the study and on the same day of the medical visit, we performed blood collection and carotid ultrasound. As a measure of disease activity, the disease activity score (DAS28) was calculated according to the ESR. Determination of swollen and tender joint counts were also obtained (TJC, SJC respectively). Pain was measured using the 0–10 visual analogue scale, and patients reported any disability with the health assessment questionnaire (HAQ) index. The DAS28 variable was categorized as remission (DAS28 < 2.6), low activity (2.6 ≤ DAS28 < 3.2), moderate activity (3.2 ≤ DAS28 ≤ 5) and high activity (DAS28 > 5.1). The Clinical Research Ethics Committee (*Comitè Ètic d’Investigació amb Medicaments)* of our hospital approved the study (reference: 11-04-28/4proj5) and informed consent was obtained from each patient. We executed the investigation in accordance with our Institution’s guidelines and the Helsinki Declaration. Patients and public were not involved in the development of the study.

### Clinical evaluation

The presence of classical CV risk factors (smoking, hypertension, diabetes and hypercholesterolemia), history of CV events and the use of hypolipidaemic, hypoglycaemic or antiplatelet drugs were collected. Additionally, joint physical examinations of RA and measurements of body weight, height, body mass index (BMI), waist circumference (WC), systolic blood pressure (SBP) and diastolic blood pressure (DBP) were taken. Determination of swollen and tender joint counts were also obtained (TJC, SJC respectively).

### Laboratory measurements

Blood samples were collected from 214 patients, who had fasted for at least 12 h. Plasma was obtained by whole blood centrifugation at 3.000 rpm for 10 min and plasma samples were stored at −80 °C for analysis. Analytical determinations included the following:

haemogram, general biochemistry, haemoglobin glycoside, thyrotropin, albumin, lipid profile [triglycerides (TG), total cholesterol (TC) and low-density lipoprotein cholesterol (LDLc), high-density lipoprotein cholesterol (HDLc) and very-low-density cholesterol (VLDLc)] performed by enzymatic methods; and rheumatoid factor (RF), citrullinated anti-cyclic peptide antibodies (ACPA), antinuclear antibodies and inflammatory markers [erythrocyte sedimentation rate (ESR), C-reactive protein (CRP) and fibrinogen] performed by conventional methods. Positive rheumatoid factor (RF +) was defined for RF values > 20 and positive citrullinated anti-cyclic peptide antibodies (ACPA +) for ACPA values > 1. Dyslipemia was defined as having HDLc < 50 mg/dL for women or < 40 mg/dL for men, or TG > 150 mg/dL, or LDLc > 100 mg/dL or in treatment with statins or other hypocholesterolemic drugs.

### Ultrasound evaluation of intima-media thickness and arterial stiffness

To measured carotid intima media thickness (cIMT), we used a My Lab 50 X-Vision sonographer (Esaote SpA, Genova, Italy) with a linear array ultrasound probe small parts broadband transducer (5–12 MHz). We identified and digitally recorded the far wall of the common carotid artery (1 cm proximal to the bifurcation), and the internal carotid artery (1 cm distal to the bifurcation) of the left and right carotid arteries. In vivo measurements of cIMT were performed at the predefined points using the QIMT© radiofrequency image processing software (Esaote SpA, Genova, Italy). To reduce observer variability, a single operator obtained and measured the images. We averaged the measurements of three static images of left and right carotid arteries to obtain the mean cIMT. We defined plaque as a focal structure encroaching into the arterial lumen by at least 0.5 mm or 50% of the surrounding IMT value, or a thickness > 1.5 mm.

Arterial stiffness expressed by the PWV and carotid distensibility was measured directly at both common carotid arteries using the ultrasound linear probe (5–12 MHz) as a tonometer and analysed in vivo by the Quality Arterial Stiffness (QAS©) radiofrequency software (Esaote SpA, Genova, Italy). The pulse wave velocity was obtained from brachial blood pressure and the accurate measurements of diameter and change in diameter of carotid arteries. Carotid distensibility was the change in diameter of the carotid artery secondary to intravascular volume expansion caused by the left ventricle systole. Vascular stiffness parameters were calculated after calibration for blood pressure^51,52^ and final values were the median measurements of the right and left carotid arteries^53,54^.

### Plasma microRNA expression

Selected miRs from the discovery study (miR Let-7a, miR-96, miR-381, miR-425-5p, miR-451, and miR-572) were validated in independent plasma samples from 214 RA patients (validation cohort). Before RNA extraction, an aliquote of 200 µl was used for hemolysis evaluation. Hemolysis was discarded after spectrophotometer analysis at λ = 414 nm, corresponding to oxy-hemoglobin contamination.

The extraction of RNA containing the fraction of small RNAs was carried out from 200 µl of frozen plasma by means of the commercial miRCURY RNA Isolation Kit (Exiqon) and following the manufacturer's instructions. Before the extraction, 1 µL of a mixture of synthetic RNAs (UniSp2, UniSp4, and UniSp5) (Exiqon) were spike in the plasma in order to control for the efficiency of the RNA extraction. In addition, 1.25 µL of MS2 RNA carrier (Roche) was added to improve RNA extraction. The final RNA extracted was eluted in 50 µL of treated water. Reverse transcription (RT) was carried out from 2 µL of the RNA obtained in a final volume of 10 µL, using miRCURY LNA Universal RT microRNA PCR and Universal cDNA synthesis kit II (Exiqon, Denmark). The conditions for the RT reaction were: incubation for 60 min at 42 °C, heat inactivation for 5 min at 95 °C, and cooling at 4 °C. The efficiency of the RT reactions were controlled by adding 0.5 µL of cel-miR-39-3p and UniSp6 (Exiqon). The resulting cDNA was diluted 1:40 before quantification by quantitative PCR (qPCR). miRNAs candidates were validated by qPCR using commercial miRCURY LNA Universal RT microRNA PCR, ExiLENT SYBR Green master mix Kit (Exiqon, Denmark) and commercial primers for each miR (hsa-miR LNA™ PCR primer set, UniRT). The qPCR amplification reactions were performed on the 7900HT Fast Real-Time PCR System (Applied Biosystems) with the following conditions: 10 min at 95 °C and 40 cycles of 10 s at 95 °C and 1 min at 60 °C. Melting curve analysis were performed to control the specificity of the qPCR.

The cycle threshold (Ct) for each sample and each miR was obtained with SDS v2.3 software (Applied Biosystems). miR-16-5p was choose as reference for normalization as showed an optimal stability after evaluation with RefFinder^55^. The relative expression of each miR in each sample was calculated using the variable ΔCt, obtained as Ct miR candidate—Ct miR-16-5p. An increase in the ΔCt variable of a particular miR represented a decrease in the expression of that miR.

### Statistical analysis

Continuous variables are presented as the mean (standard deviation), and categorical variables are presented as the percentage (number of individuals). ANOVA was used to evaluate differences between groups followed by Bonferroni correction as a post hoc test. For categorical variables, the differences between the proportions were analysed using the chi-Squared test. Bivariate correlations were estimated using the Pearson correlation coefficient “r”. To evaluate miR associations with dependent variables (cIMT, PWV, and distensibility), multiple linear regression was used with multivariate models. Because in a previous study to estimate the cIMT variable, we reported a significant interaction between age and sex^18^, in the present study, multivariable models for cIMT associations had to be performed with the population stratified into men and women. The R-squared (R^2^) statistic was used to provide an estimate of the percentage of the response variable variability that was explained by a linear model. The heterogeneity of the RA cohort in terms of disease activity, inflammatory features and treatments was assessed by interaction analyses. Interaction terms between DAS28, CRP, and RA treatments (corticosteroid, DMARDs, biological agent, and NSAIDs) and DCt425 and DCt425 were added to the linear regression models. The interaction terms were considered significant when the variability of the dependent variable explained by the model significantly improved.

Multivariate logistic regression was used to estimate the presence of carotid plaques. RA patients with prior cardiovascular disease and those with a cIMT above the 75th percentile were binarily categorized into variables named cardiovascular disease (CVD) and pathological cIMT (pat-cIMT), respectively. To evaluate the association between these variables and miR-425-5p and miR-451 expression levels, stepwise logistic multivariable regression models were adjusted with or without the addition of miR variables. The receiver operating characteristic (ROC) curves and area under the ROC curve (AUC) values were calculated as a measure of the classification accuracy of each adjusted model. Significance between the models was evaluated by Akaike information criteria (AIC) and the likelihood ratio test (LR). We estimated the best cut-off value and the sensitivity, specificity, positive predictive value (PPV) and negative predictive value (NPV) for the optimal cut-off point by means of the Youden index. Furthermore, we performed a random forest (RF) analysis based on conditional inference trees in which 1000 trees were grown to evaluate the importance of each variable in the decision method, which was represented in terms of the mean decrease Gini plot*.* In multivariable regression analysis, we initially selected clinically relevant variables and known confounders for inclusion in the models. Furthermore, the iterative process of variable selection was performed using stepwise linear and logistic regression analyses. A *p*-value of < 0.05 was considered statistically significant.

Biological functional analyses of both miR-425-5p and miR-451 were performed. First, we predicted gene targets of each miRNA using mirDIP v1.4, which is an integrative database for human miRNA target predictions. MirDIP provides nearly 152 million human miRNA-target predictions, which were collected across 30 different resources^56^. We selected the top 5% prediction targets of each miRNA according to the integrative score. Second, we performed a functional enrichment analysis using G:Profiler. Gene Ontology (GO) terms and pathways from the Kyoto Encyclopedia of Genes and Genomes (KEGG) database were identified in the target gene list of each miRNA. G: Profiler uses multiple testing corrections and applies the tailor-made algorithm G: SCS to reduce significance scores^57^. The GO results are shown according to molecular function and biological process, and the KEGG pathways^58^ are presented. Each GO term and pathway is showed with its adjusted *p* value. Statistical software SPSS, version 23 and R Studio, version 3.6 were used to analyse the data.

## Supplementary Information

Supplementary Information.

## Data Availability

The datasets analysed during the current study are available from the corresponding author on reasonable request.
