# Supplementary material for: Plasma expression of microRNA-425-5p and microRNA-451a as biomarkers of cardiovascular disease in rheumatoid arthritis patients
Source: Sci Rep. 2021 Aug 2;11:15670. doi: 10.1038/s41598-021-95234-w (PMC8329234; doi:10.1038/s41598-021-95234-w)

**Plasma expression of microRNA-425-5p and microRNA-451a as biomarkers of cardiovascular disease in rheumatoid arthritis patients**

Delia Taverner, Dídac Llop, Roser Rosales, Raimon Ferré, Luis Masana, Joan-Carles Vallvé, Silvia Paredes

**Supplementary Materials**

**Supplementary Table 1:** Univariate correlations of the candidate miRs with cIMT stratified by sex

|  | cIMT | | | |
| --- | --- | --- | --- | --- |
|  | Female  n=138 | | Male  n=76 | |
|  | r | p | r | p |
| miR-Let7a | 0.049 | 0.288 | 0.057 | 0.318 |
| miR-96 | -0.009 | 0.463 | 0.1 | 0.208 |
| miR-381 | 0.032 | 0.411 | 0.058 | 0.396 |
| miR-425-5p | 0.051 | 0.280 | 0.122 | 0.153 |
| miR-451 | -0.157 | 0.035 | -0.023 | 0.427 |
| miR-572 | -0.04 | 0.398 | -0.135 | 0.228 |

R = Pearson’s coefficient, p = p value, cIMT = carotid intima-media thickness.

**Supplementary Table 2:** Age-adjusted β regression estimates of the effect of candidate miR expression on cIMT stratified by sex obtained with linear regression analyses

|  | Female | | | | | Male | | | | |
| --- | --- | --- | --- | --- | --- | --- | --- | --- | --- | --- |
|  | β | SE | 95% CI | | p | β | SE | 95% CI | | p |
|  |  |  | Lower | Higher |  |  |  | Lower | Higher |  |
| miR-Let7a | 0,009 | 0,008 | -0,008 | 0,02 | 0.303 | 0,012 | 0,013 | -0,013 | 0,038 | 0.325 |
| miR-96 | -0,003 | 0,005 | -0,012 | 0,007 | 0.601 | 0,007 | 0,008 | -0,009 | 0,022 | 0.415 |
| miR-381 | -0,001 | 0,005 | -0,01 | 0,01 | 0.86 | 0,009 | 0,01 | -0,014 | 0,03 | 0.45 |
| miR-425-5p | 0,02 | 0,02 | -0,02 | 0,054 | 0.37 | 0,052 | 0,03 | 0,001 | 0,103 | 0.045 |
| miR-451 | -0,046 | 0,02 | -0,08 | -0,008 | 0.019 | -0,021 | 0,03 | -0,07 | 0,03 | 0.44 |
| miR-572 | -0,005 | 0,008 | -0,02 | 0,01 | 0.57 | -0,001 | 0,007 | -0,02 | 0,01 | 0.85 |

SE= standard error, β = linear regression estimates, CI = confidence interval.

**Supplementary Figure 1:** Percentage of cIMT variability (measured with the R-squared statistic) that is accounted for by the different multivariate linear models applied to men and women separately. The regression model 1 included the following variables: RA disease onset, body mass index, age, ischaemic heart disease, ictus, peripheral artery disease, creatinine, hypertension, dyslipidaemia, type 2 diabetes mellitus, disease-modifying antirheumatic drugs, biological agents, corticosteroids, and non-steroidal anti-inflammatory drugs. Model 2 included variables from model 1 plus miR-425-5p expression in men and miR-451 expression in women.


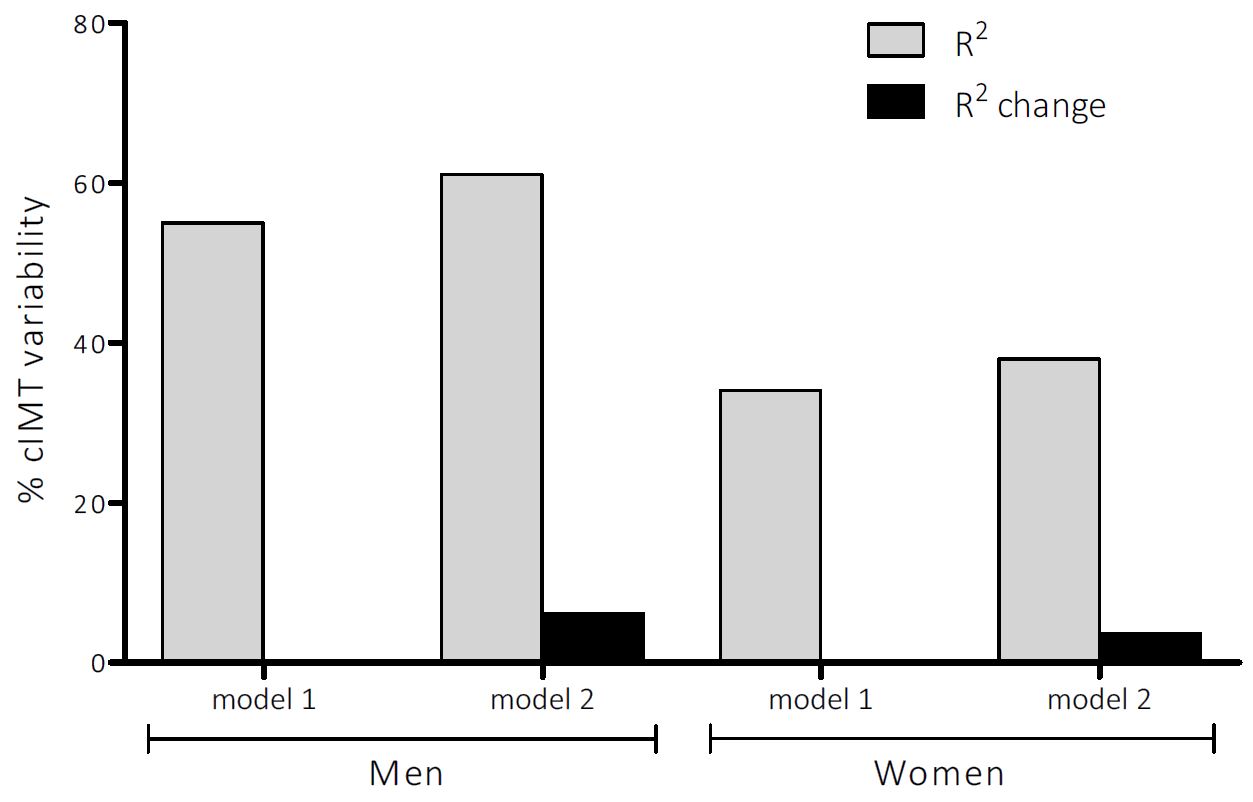


**Supplementary figure 2:** Correlation between observed cIMT and the cIMT predicted by the model for men (A) and women (B)

A)
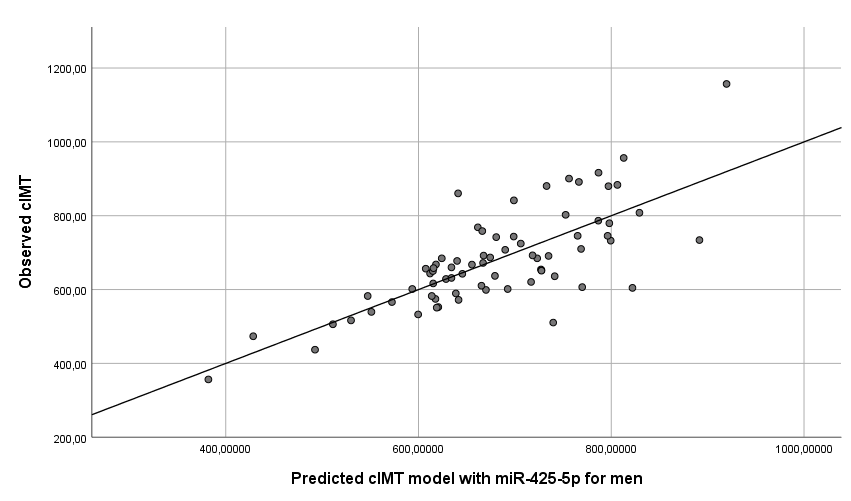


B)


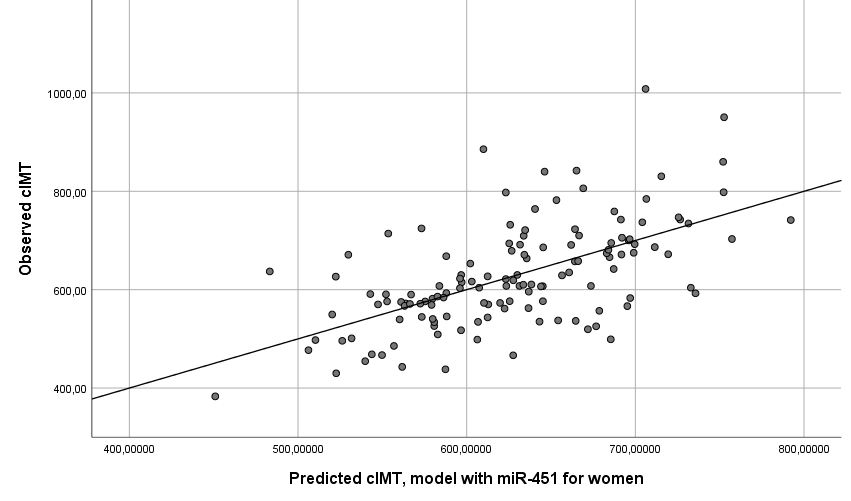


**Supplementary table 3:** Interaction terms from the multivariable linear regression models between miR-425-5p and miR-451 and disease activity, inflammatory characteristics and treatment variables. The models were applied to estimate cIMT and PWV.

| **cIMT** | | | | | |
| --- | --- | --- | --- | --- | --- |
| **Men** | | | | | |
| **Interaction term** | **β** | **SE** | **p-value** | **95% CI** | |
| 425*DAS28 | 0,01 | 0,023 | 0,63 | -0,036 | 0,06 |
| 425*RF+ | 0,0027 | 0,05 | 0,96 | -0,1 | 0,11 |
| 425*DLP | -0,004 | 0,05 | 0,93 | -0,11 | 0,1 |
| 425*CRP | 0,032 | 0,03 | 0,37 | -0,038 | 0,1 |
| 425*Corticosteroids | 0,06 | 0,06 | 0,34 | -0,064 | 0,185 |
| 425*DMARDs | 0,09 | 0,09 | 0,35 | -0,1 | 0,28 |
| 425*NSAIDs | 0,0038 | 0,05 | 0,94 | -0,1 | 0,11 |
| 425*Biological agent | 0,0075 | 0,115 | 0,95 | -0,22 | 0,23 |
| **Women** | | | | | |
| **Interaction term** | **β** | **SE** | **p-value** | **95% CI** | |
| 451*DAS28 | 0,0008 | 0,015 | 0,956 | -0,028 | 0,03 |
| 451*RF+ | 0,004 | 0,045 | 0,93 | -0,086 | 0,0945 |
| 451*DLP | -0,06 | 0,04 | 0,13 | -0,14 | 0,018 |
| 451*CRP | -0,007 | 0,24 | 0,76 | -0,055 | 0,04 |
| 451*Corticosteroids | 0,06 | 0,04 | 0,1 | -0,013 | 0,14 |
| 451*DMARDs | -0,04 | 0,02 | 0,38 | -0,13 | 0,05 |
| 451*NSAIDs | 0,025 | 0,042 | 0,55 | -0,06 | 0,004 |
| 451*Biological agent | -0,023 | 0,048 | 0,62 | -0,11 | 0,07 |
| **PWV** | | | | | |
| **Overall population** | | | | | |
| **Interaction term** | **β** | **SE** | **p-value** | **95% CI** | |
| 451*DAS28 | 0,021 | 0,012 | 0,07 | -0,0018 | 0,045 |
| 451*RF+ | 0,046 | 0,031 | 0,12 | -0,016 | 0,11 |
| 451*DLP | 0,056 | 0,03 | 0,08 | -0,002 | 0,11 |
| 451*CRP | 0,03 | 0,02 | 0,1 | -0,006 | 0,07 |
| 451*Corticosteroids | 0,015 | 0,03 | 0,61 | -0,044 | 0,07 |
| 451*DMARDs | -0,05 | 0,036 | 0,18 | -0,12 | 0,022 |
| 451*NSAIDs | 0,021 | 0,03 | 0,47 | -0,04 | 0,08 |
| 451*Biological agent | 0,009 | 0,04 | 0,81 | -0,07 | 0,09 |

RF+= positive rheumatoid factor, DLP = dyslipidaemia, CRP = C reactive protein, cIMT = carotid intima-media thickness, PWV = pulse wave velocity, SE = standard error, β = linear regression estimates, CI = confidence interval. DMARDs = disease-modifying antirheumatic drugs, NSAIDs = non-steroidal anti-inflammatory drugs.

**Supplementary table 4:** Adjusted OR estimates of the effect of miR expression on carotid plaque presence. Models were adjusted for age, smoking, HAQ, ESR, and RF+ and applied to the overall population. OR = odds ratio, HAQ = heath assessment questionnaire index, ESR = erythrocyte sedimentation rate, and RF+ = positive rheumatoid factor.

|  | Carotid plaque presence | | | |
| --- | --- | --- | --- | --- |
|  | OR | 95% CI | | P value |
| miR-Let7a | 0.82 | 0.56 | 1.18 | 0.3 |
| miR-96 | 1.11 | 0.91 | 1.36 | 0.3 |
| miR-381 | 1.02 | 0.77 | 1.34 | 0.88 |
| miR-425-5p | 0.97 | 0.44 | 2.14 | 0.9 |
| miR-451 | 1.28 | 0.57 | 2.85 | 0.55 |
| miR-572 | 0.71 | 0.52 | 0.1 | 0.08 |

**Supplementary figure 3:** Random forest variable relative importance for the prior CVD model, DLP = dyslipidemia, DMT2 = diabetes mellitus type 2, NSAIDs = non-steroidal anti-inflammatory drugs.


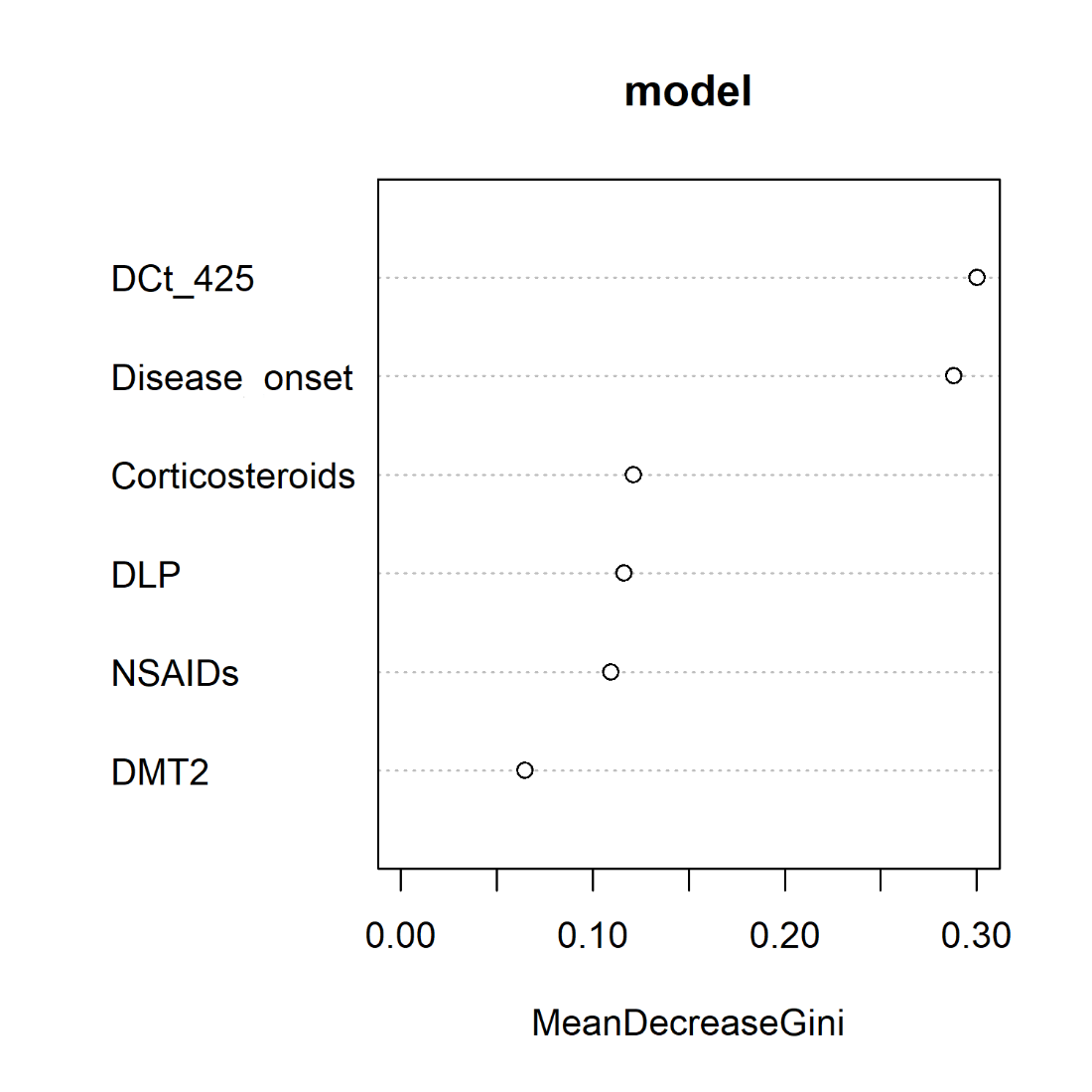

Supplement: Supplementary file 1 — Supplementary Information. [file 41598_2021_95234_MOESM1_ESM.docx]
